# Seed classification with random forest models

**DOI:** 10.1002/aps3.11596

**Published:** 2024-06-05

**Authors:** Josephine Elena Reek, Janneke Hille Ris Lambers, Eléonore Perret, Alana R. O. Chin

**Affiliations:** ^1^ Institute of Integrative Biology, ETH Zürich Zürich Switzerland; ^2^ Department of Biological Sciences California State Polytechnic University, Humboldt, Arcata California USA

**Keywords:** automated identification, forest monitoring, random forest, seed classification, seed trap

## Abstract

**Premise:**

To improve forest conservation monitoring, we developed a protocol to automatically count and identify the seeds of plant species with minimal resource requirements, making the process more efficient and less dependent on human operators.

**Methods and Results:**

Seeds from six North American conifer tree species were separated from leaf litter and imaged on a flatbed scanner. In the most successful species‐classification approach, an ImageJ macro automatically extracted measurements for random forest classification in the software R. The method allows for good classification accuracy, and the same process can be used to train the model on other species.

**Conclusions:**

This protocol is an adaptable tool for efficient and consistent identification of seed species or potentially other objects. Automated seed classification is efficient and inexpensive, making it a practical solution that enhances the feasibility of large‐scale monitoring projects in conservation biology.

## Abstrakt

**Prämisse:**

Um Schutz und Monitoring von Wäldern zu unterstützen, haben wir ein Protokoll für automatisches Zählen und Identifizieren von Pflanzensamen entwickelt. Es erfordert minimale Ressourcen und bietet Zeit‐Effizienz wie auch geringe Abhängigkeit vom Anwender.

**Methoden und Resultate:**

Samen von sechs Nordamerikanischen Koniferen‐ Arten wurden von Laubstreu getrennt und auf einem Flachbettscanner gescannt. Die erfolgreichste Klassifikationsmethode benutzt ein ImageJ macro um Messwerte automatisch zu extrahieren; diese werden zur Klassifikation durch ein Random Forest Modell in der Software R verwendet. Die Methode bietet gute Klassifikationsgenauigkeit und derselbe Prozess kann benutzt werden, um das Modell auf andere Arten zu trainieren.

**Schlussfolgerung:**

Das Protokoll ist ein anpassungsfähiges Mittel um Samenarten oder potentiell andere Objekte effizient und einheitlich zu identifizieren. Automatische Samenklassifikation ist effizient und kostengünstig und somit eine praktikable Möglichkeit für grosse Monitoring Projekte im Naturschutz.

## Abstrait

**Résumé:**

Afin d'améliorer la surveillance des écosystèmes forestiers, nous avons développés une nouvelle méthode pour le comptage et l'identification des graines de plantes en fonction des espèces. En utilisant des ressources minimales, ce protocole permet de gagner du temps et de réduire la nécessité d'intervention humaine constante.

**Méthodes et Résultats:**

Après avoir été séparées de la litière de feuilles, les graines de six espèces de conifères d'Amérique du Nord ont été photographiées sur un scanner. Lors de l'approche de classification des espèces la plus réussie, une macro ImageJ extrait des mesures pour la classification par forêt aléatoire dans le logiciel R. La méthode présente une précision élevée de classification et le processus peut être adapté pour entraîner le modèle sur d'autres espèces.

**Conclusion:**

Ce protocol est un outil extrêmement polyvalent pour une identification efficace et cohérente des espèces de semences, ou potentiellement d'autres objets. La classification automatisée des graines est à la fois efficace et peu coûteuse, ce qui en fait une solution pratique pour améliorer la réalisation de projets à grande échelle dans le domaine de la biologie de la conservation.

Long‐term monitoring projects are an important component of conservation biology, allowing us to detect changes in important organismal performance measures over time, such as seed production in forest systems (Loiselle et al., 1996; McEuen and Curran, 2004; Reid et al., 2015; FAO, 2020). The classification of plant seeds by species in long‐term monitoring projects is a common task in diverse areas of biological and agricultural sciences and practices (Clark et al., 2021; Mathesius, 2021; Qiu et al., 2022); however, the difficulty of manual seed identification requires extensive work from a skilled technician, which limits the wider use of seed‐trap monitoring. Seed classification tasks include physically separating the seeds, determining the presence or absence of certain species, and counting the number of seeds that are present for a given selection of species. In the present study, we developed a protocol (Appendix 1) to make the latter, most difficult, task of counting seeds per species more efficient, although there are other potential applications.

Two main methods are currently employed for counting unknown numbers and identities of species in a sample of seeds. One is the use of sorting machines, which must be trained to the specific seed species in question before they physically separate the seeds in a binary manner. This means multiple rounds are required if more than two species are to be identified and counted, as only one species can be separated out per run (species in question: yes or no). Sorting machines can reach high accuracy, but they are expensive, require effort to use, and are not free of error (e.g., Shulgin et al., 2014; Bracacescu et al., 2018; Kanjanawanishkul et al., 2018). An alternative approach is the manual counting and sorting of seeds from the sample. Manual sorting requires no technical resources but does require a substantial time investment by skilled individuals who have been trained for the task. Even then, individual variation between operators cannot be eliminated or readily quantified, especially if species have similar seeds and/or seeds have been damaged or weathered. There can also be variation within individual operators as they become more practiced at the task.

The need to remove operator error presents an important motivation to move toward automated seed identification in multi‐year monitoring projects, where technician skill and turnover could introduce bias among collection years that could confound research into interannual variation in seed production. As they have become more powerful and accessible, image‐ and computer‐based methods have been increasingly employed for seed‐counting tasks. Mussadiq et al. (2015) reviewed different programs for counting seeds within an image without differentiating between species or properties. Steinecke et al. (2023) employed an image‐based method to analyze morphological seed traits. Both of these analyses found ImageJ (Schneider et al., 2012), which our method also employs, to be a useful program. Here, we take this approach further by using the measurable morphological traits to classify the seeds into different species, using only a flatbed scanner, a standard computer, and, optionally, a drying oven. The protocol (Appendix 1) was developed on six North American conifer species (Appendix S1): *Abies amabilis* Douglas ex J. Forbes (Pinaceae), *Callitropsis nootkatensis* (D. Don) Oerst. (Cupressaceae), *Pseudotsuga menziesii* (Mirb.) Franco (Pinaceae), *Thuja plicata* Donn ex D. Don (Cupressaceae), *Tsuga heterophylla* Sarg. (Pinaceae), and *Tsuga mertensiana* (Bong.) Carrière (Pinaceae). This approach makes seed counting much faster (once isolated from larger leaf litter), as long as the potential species present are known. Pieces of remaining forest litter smaller than seeds can be automatically excluded using Fiji (Schindelin et al., 2012), an image‐processing package within ImageJ. The automated identification reduces the operating time as well as the operator training time, and the results are largely independent of the operator. Computer‐based identification is a potentially useful tool in studies using seeds to understand community composition, forest regeneration potential, or the reproductive dynamics of seed‐producing plants.

## METHODS AND RESULTS

### Premise

**Figure 1:**
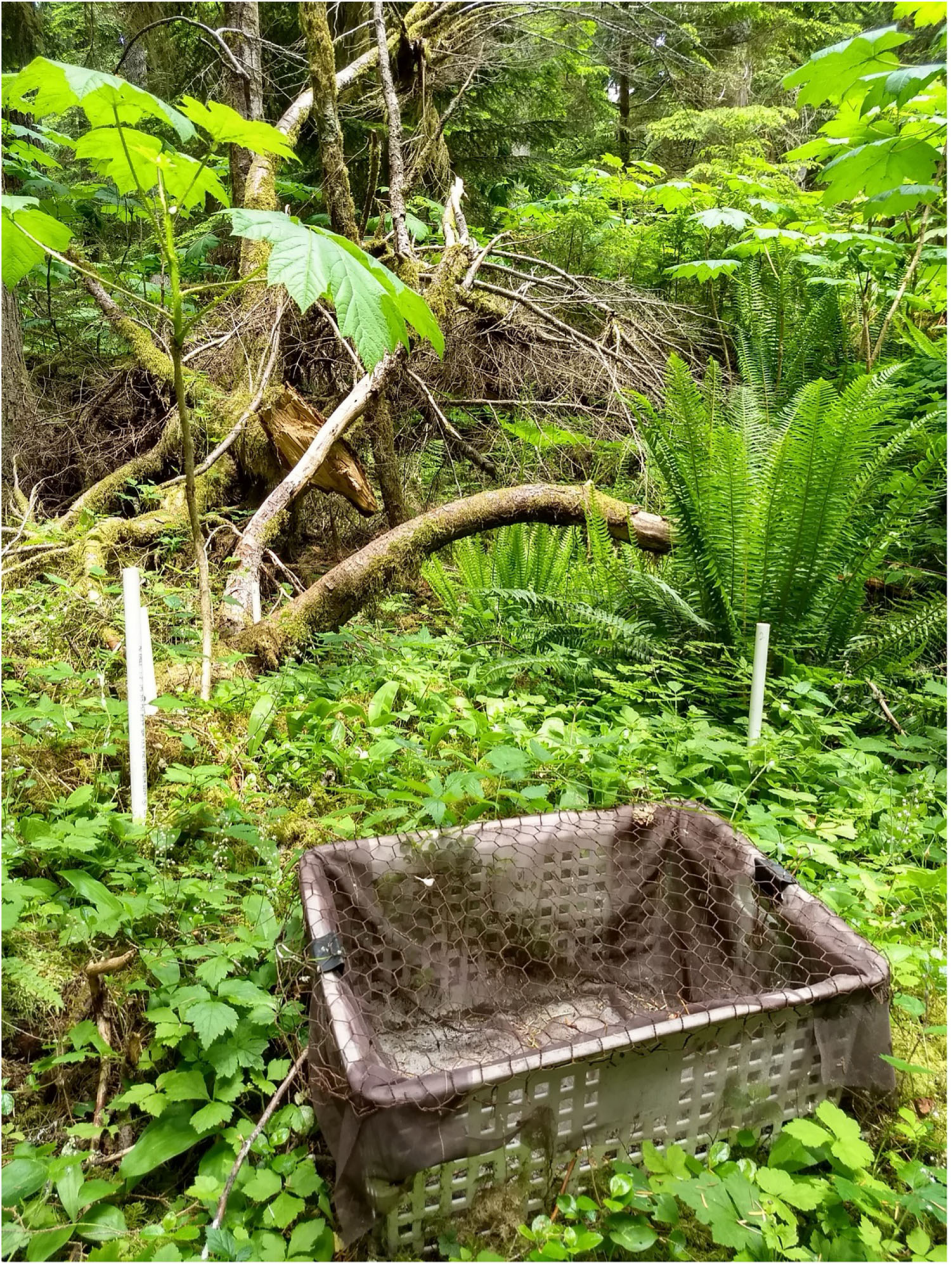
Example of a seed trap used for seed collection.

We used seeds collected in forest seed traps (Figure 1) on Mt. Tahoma, Mt. Rainier National Park, Washington, USA. This study site provided a fitting test case, as the seeds and leaf litter (needles) are similar in size and general shape and species diversity in the forests is relatively low (Kroiss and Hille Ris Lambers, 2015).

**Figure 2:**
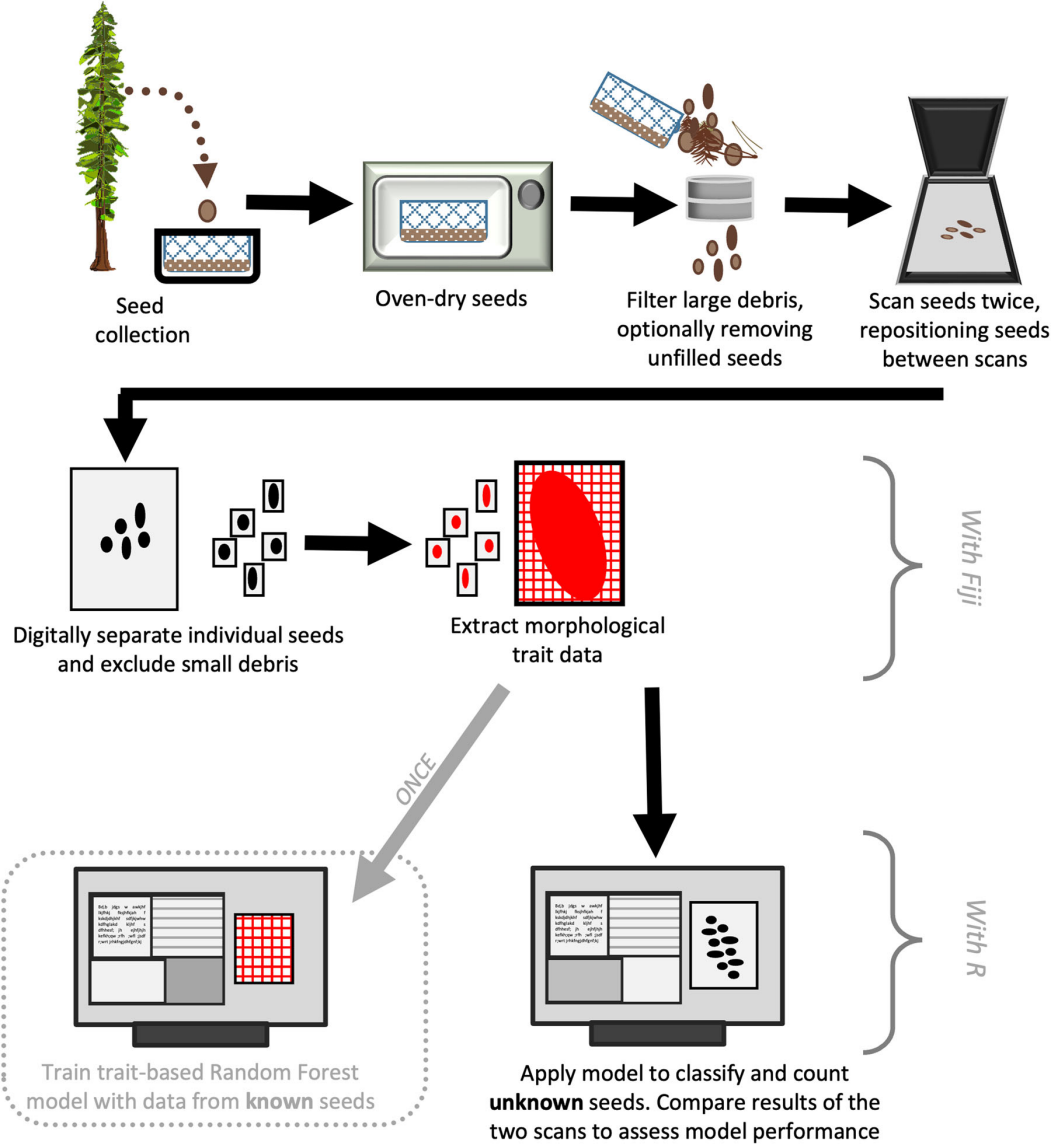
Workflow diagram. Seeds collected in the forest are dried, separated from leaf litter with soil sieves, and scanned. Morphological traits are digitally extracted with Fiji in ImageJ, and a random forest classifier model is built and applied in R.

We had two criteria for a useful protocol. First, the protocol steps should be performed efficiently, without special materials beyond what is likely to be available at research stations or small institutions, and with minimal impact of the operator on the output (ensuring low user error). Second, the method should be reliable even if seeds are weathered or in contact with lichen and fungi while in the seed traps. To avoid lichen‐induced color bias, the analyses were mainly based on black‐and‐white images from a flatbed scanner, although color versions were also explored and code for their implementation is provided. The full workflow is outlined in Figure 2.

### Seed collection and preparation

Seeds were collected in seed traps (Figure 1) and labeled by seed trap and collection date. Seeds were dried for 24 h at 40°C and separated from the leaf litter using a 1‐mm soil sieve. If filled and unfilled seeds are to be distinguished, they must be separated in this step (details of different approaches [e.g., floating ability] are provided in Appendix 1 under Step 1b). The seeds are then labeled and treated as separate batches for the analysis. The training and test data came from seeds that were hand‐sorted into species by the same experienced individual. Both filled and unfilled seeds were included and handled in separate batches. They belonged to six species, the seeds of which show varying levels of distinguishing features (examples in Table 1): *Abies amabilis*, *Callitropsis nootkatensis*, *Pseudotsuga menziesii*, *Thuja plicata*, *Tsuga heterophylla*, and *Tsuga mertensiana*. The two *Tsuga* species were very difficult to distinguish visually, although all six species (being conifers) are quite similar and take skill to recognize.

**Table 1:** Example scans of the seeds of the species used (two per species).

ABAM	CANO	PSME	THPL	TSHE	TSME
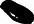	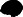	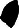	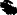	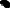	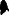
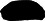	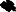	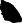	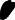	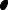	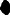

*Note*: ABAM = *Abies amabilis*; CANO = *Callitropsis nootkatensis*; PSME = *Pseudotsuga menziesii*; THPL = *Thuja plicata*; TSHE = *Tsuga heterophylla*; TSME = *Tsuga mertensiana*.

### Imaging and digital image processing

The (previously identified) seeds of each species from each sample (filled or unfilled seeds from one seed trap) were scanned separately. Each of the 554 scans contained one to approximately 90 seeds (*N* = 2874 seeds from 59 traps and 10 sites; details in Appendix S2). We scanned each batch of seeds twice, moving the seeds around and/or flipping them over between scans to capture different potential angles; these scans were labeled as replicates of the same seeds. Both replicate arrangements of the same seeds were scanned as TIFF files in black‐and‐white mode at 600 dpi (training and test batches of seeds need to be scanned with the same dpi). The scanner produced binary black‐and‐white images directly (no grayscale). The scans were imported into Fiji in ImageJ (Schindelin et al., 2012), where individual seeds were delimited by thresholding to remove the background and create individual binary seed selections. We set a lower limit on object size so that debris smaller than the seeds would not be counted as an object to be measured. For the measurement‐based classification, an ImageJ macro (available from https://github.com/JosephineEReek/Seed-classification-with-random-forest-models; see Data Availability Statement) was written to extract morphological measurements (e.g., area, circumference, circularity, Feret diameter; details in Table 2) from each seed in the scan (Figure 2). We recommend the use of all available traits in Fiji in ImageJ, as even potentially low‐importance traits may increase model reliability without adding any work for the user.

The variety of measurements built robustness against seed damage. If a part breaks off the side of a seed or its wing, for example, the Feret diameter may remain unchanged, and a large seed with a broken wing may still have a large area. Having two scans per batch also reduces the influence of seed damage, as seeds are more likely to land in an orientation where the damage is less influential in the two‐dimensional images. Nevertheless, seed damage makes classification more difficult and imprecise. This would be most influential if the training data came from pristine seeds (in our case it did not); training seeds should have been exposed to the same weathering as the test sample so that typical forms of damage (e.g., broken wings) will be incorporated into the model.

### Seed species classification

The extracted measurements of known seeds were imported into R for model‐based classification (Figure 2). For each species, we randomly allocated 80% of the seeds to the training set and 20% to the test set. The chosen random forest (RF) model was implemented in R version 4.2.2 (R Core Team, 2022) and RStudio version 2022.07.2 (RStudio Team, 2020), using the packages randomForest (Liaw and Wiener, 2002) and caret (Kuhn, 2016). In RF, a number of decision trees are computed, with each node using a subset of the training samples and the variables provided. Each tree used one variable at each branching point to send the input down one or the other branch. At the end of the branches, it was assigned to a class based on the path it took. In the classification task, an input (in this case, all morphological measurements of one seed) was run through all of the established trees and the class (i.e., species) predicted by the most trees was assigned to the input seed (Hastie et al., 2009). Each individual seed is classified, then the total seed trap content is summarized. Because we scanned each batch of seeds twice, the results of the analyses could then be compared to determine a consensus “mean” classification and calculate the discrepancy between the scans. This discrepancy is reported in absolute terms (number of seeds that are different), as well as in relative terms (number of seeds that are different standardized by the number of seeds on the scan). The comparison of multiple scans allows for more robustness and is especially helpful when seeds are damaged or when debris is scanned together with the seeds. Alternatively, the process can be easily adapted to save time by using only one scan per batch, or more than two scans of each batch could be compared in cases where there is more debris or where the seeds tend to balance on their sides.

### Validation

The chosen RF model uses 500 trees and tries five variables per split (see Appendices S3 and S4 for the other methods explored), using the predictor as detailed in Table 1 and with species as the response variable. The model was tuned for the parameters “ntree” (number of trees in the RF) and “mtry” (number of variables randomly sampled as candidates at each split); however, we did not observe major changes in performance when these variables were changed (details in Appendix S4). In adapting this protocol (Appendix 1) to new species and sites, it is likely that a different combination of variables may provide better results. If no variables are specified, the RF function will default to an estimate, which is a good starting point from which to experiment. The overall accuracy of our method was assessed using the model statistics presented in Table 3. In addition to a percent accuracy, we also calculated the kappa statistic, which compares the observed accuracy of the model to an expected accuracy that would be reached by a random classifier. On the training data set, the RF model reached a full accuracy of 100% (95% confidence interval [CI] = 0.9992, 1; kappa = 1, *n* = 4889). On the test data set, the RF model reached an accuracy of 93% (95% CI = 0.9123, 0.9473; kappa = 0.8887; *n* = 859). To determine how the model performed on the different species, we computed confusion matrices (Appendix S5). This table lists the actual species in rows and the model‐predicted species in columns. It is thus possible to see which pairs of species were most frequently confused. In our case, the mispredictions leading to non‐perfect accuracy came mostly from the *Thuja* and *Tsuga* species. The importance of the different parameters in predicting species can be assessed with a Gini coefficient. The mean decrease in Gini (Appendix S5) represents how much the classification impurity is reduced by the nodes of the variable in question. We determined the site to have a high impact on classification accuracy, probably related to species occurrence patterns or morphological shifts along our large elevational gradient, while variables relating to seed size (area, perimeter, Major, Minor, Feret diameter, and IntDen; see Table 2 for variable definitions) also had an important impact. Running the RF model with only nodes with an importance over 50 marginally decreased the prediction accuracy to 92.32% for the test data.

**Table 2:** Measurements extracted from Fiji in ImageJ and used in the random forest model. "Selection" refers to the seed itself, not a rectangular box of the background including the seed. Details available in the ImageJ user manual at https://imagej.nih.gov/ij/docs/guide/146-30.html#sub:Set-Measurements. An additional model excluding *xy*‐type predictors (X, Y, XM, YM, BX, BY, FeretX, FeretY) is presented in Appendix S4.

Measurements taken from black‐and‐white scans	Definition
Area	Area of selection in calibrated square units
X	The centroid of the selection (*x*‐coordinate)
Y	The centroid of the selection (*y*‐coordinate)
XM	Center of mass. The brightness‐weighted average of the *x*‐coordinates of all pixels in the selection. First‐order spatial moments.
YM	Center of mass. The brightness‐weighted average of the *y*‐coordinates of all pixels in the selection. First‐order spatial moments.
Perim.	The length of the outside boundary of the selection
BX	The *x*‐coordinate of the upper left corner of the smallest rectangle enclosing the selection
BY	The *y*‐coordinate of the upper left corner of the smallest rectangle enclosing the selection
Width	Width of the smallest rectangle enclosing the selection
Height	Height of the smallest rectangle enclosing the selection
Major	Fits an ellipse to the selection. Primary axis of the best‐fitting ellipse.
Minor	Fits an ellipse to the selection. Secondary axis of the best‐fitting ellipse.
Angle	Fits an ellipse to the selection. Angle between the primary axis and a line parallel to the *x*‐axis of the image.
Circ.	Circumference: *4π × [Area]* *[Perimeter]* ^*2*^ with a value of 1.0 indicating a perfect circle.
Feret diameter	The longest distance between any two points along the selection boundary, also known as maximum caliper
IntDen	Integrated density. The sum of the values of the pixels in the selection × the area of one pixel (different from RawIntDen only if the image is scaled, which these are).
Median	The median value of the pixels in the selection
Skew^a^	The third‐order moment about the mean
Kurt^a^	The fourth‐order moment about the mean
RawIntDen	Raw integrated density. The sum of the values of the pixels in the selection.
FeretX	Starting *x*‐coordinate of the Feret diameter
FeretY	Starting *y*‐coordinate of the Feret diameter
FeretAngle	The angle (0–180°) of the Feret diameter
MinFeret	Minimum caliper diameter
AR	The aspect ratio of the particle's fitted ellipse, i.e., [*Major Axis]/[Minor Axis]*
Round	4 × [*Area*]/*π* × [*Major axis*]^2^ or the inverse of *Aspect Ratio*
Solidity	[*Area]/[Convex Area]*
Site^b^	Study site at which the seed was collected

^a^ Measured using the Fiji in ImageJ macro, but not used in our version of the model due to frequent missing values. This could be included in a system in which these measurements do not result in missing values.

^b^ This is not measured by the macro but extracted from the “tag” column in the macro's output file, which tracks the name of the scanned TIFF file from which the measurements are taken. That file name should include collection site information as per the protocol (Appendix 1).

**Table 3:** Model statistics of the random forest models.

Statistic	Training data	Test data
Number of trees	500	500
Number of variables tried at each split	5	5
Out‐of‐bag estimate of error rate	6.93%	6.93%
Accuracy	1	0.9313
95% confidence interval of accuracy	(0.9992, 1)	(0.9123, 0.9473)
No information rate	0.4972	0.4994
Kappa	1	0.8887
Size of data set	4889 observations	859 observations

### Comparison with the current manual method

We have previously relied on the manual identification of individual seeds. A major advantage of our new automated method is the much lower operator dependency of the resulting classification, leading to better reproducibility. This is especially important in long‐term monitoring projects, as technicians usually become increasingly experienced at the task, improving their skills and potentially changing their seed characterization over the years. Apart from general factors, such as the amount of seeds and litter, the time required for the manual task depends largely on the experience of our technicians, and the level of accuracy is hard to determine and likely also heavily dependent on skill level. We estimated that the accuracy of our model‐identification approach is ~2–5% lower than manual identification (based on how often different seed sorters have to go with a “best guess” in species identification); however, now that the technique is established, we can improve our model accuracy with a much larger training set obtained from nursery seeds. Directly comparing in this way would allow for greater certainty in the correct classification of training and test data than is typical with current human‐sorted materials and should be considered for systems where a precise time and certainty estimate is important. Gains in efficiency depend, among other factors, on the number of seeds per trap and whether filled and unfilled seeds have to be distinguished. We estimate the RF model–based approach is 4–5× faster than can be achieved by our technicians, leading to large cost savings, as well as creating new opportunities for expanded sampling efforts.

## CONCLUSIONS

The method described here facilitates seed classification with reasonable accuracy (only 2.5% lower than that of manual identification) and higher efficiency than manual identification. Operator dependency and training time are reduced in comparison with manual sorting, while the material costs are much lower than those required for a sorting machine. In addition to costing up to US$100,000, advanced sorting machines can have a footprint larger than 4 × 1.5 m and non‐optionally rely on color, which can be problematic when using field‐collected seeds. Our protocol (Appendix 1) is applicable to most co‐occurring species with scannable seeds (or other non‐seed objects fulfilling this requirement). Because it is a classification task, the possible outcome (i.e., species present at the sampling site) must be known in advance and used to train the algorithm. The size of the training data set required depends on the number and similarity of the species present. There is a general limitation in RF models when the number of classes (i.e., species; here six) comes too close to the number of features used in the prediction (here 26), which should be considered before application. The protocol can also be easily adapted to include other predictors (e.g., season or color; see the example using color in Appendix S3 and the accompanying code) that might be relevant to a particular study system.

In conclusion, we believe that this will be a useful tool for ecological monitoring studies, especially when the availability and training of skilled staff to count and sort seeds are limited and seeds are sampled over a period of years where year‐to‐year accuracy is important. The need for seed identification is common in many types of conservation biology studies that investigate plant responses to climate change, such as forest regeneration, species migration, scat analysis, grassland ecology, or changes in community composition. Efficient and reliable sample processing, such as our protocol can provide, allows these projects to operate on large scales and over long periods of time, enhancing the conclusions that can be drawn from the data.

## AUTHOR CONTRIBUTIONS

This study was designed by J.E.R. and A.R.O.C., the analysis was performed by J.E.R., and the manuscript was written by J.E.R. with input from A.R.O.C. The protocol was tested by E.P., who also provided information on manual sorting. E.P. and J.H.R.L. provided data and revised the manuscript. All authors approved the final version of the manuscript.

## Supporting information


**Appendix S1.** Species names and abbreviations.
**Appendix S2.** Sample size.
**Appendix S3.** Additional classification approaches explored.
**Appendix S4.** Random forest model parameter tuning.
**Appendix S5.** Confusion matrices and node importance of the black‐and‐white model.

## Figures and Tables

**Figure A1:**
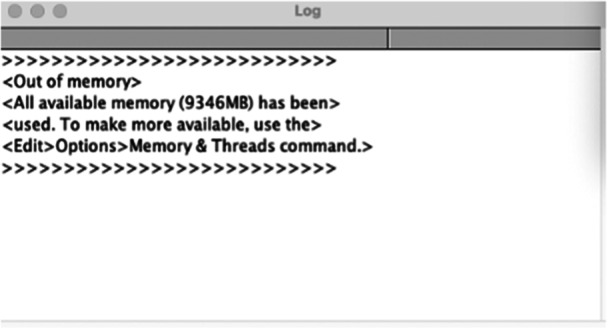
“Out of memory” error message in ImageJ. If this message appears, proceed as described in step 3c.

**Figure A2:**
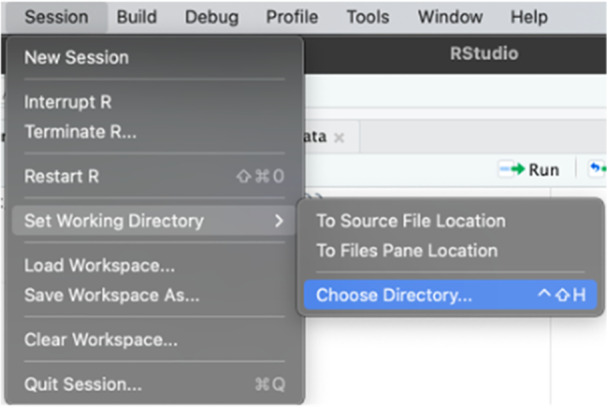
Path to set a working directory in RStudio.

## Data Availability

All code and necessary training data set are available from GitHub: https://github.com/JosephineEReek/Seed-classification-with-random-forest-models.

## Appendix 1 Protocol to automatically count and identify the seeds of plant species.

Note: This protocol describes the black‐and‐white version. Information on running the color version or adapting the protocol to new species and sites can be found below.

**Equipment:**

- Scanner: CanoScan LiDE 220 (Canon, Tokyo, Japan)
- Any computer that can run the programs detailed below
- Optionally: drying oven, 1‐mm soil sieve

**Before first use on a device:**

- Save the folder “Seed_Analysis” (available on GitHub; see Data Availability Statement) with all the necessary files to the desktop. Create an empty folder inside it called “Scans”.
- Make sure all necessary programs are installed:
  ○ Canon IJ Scan Utility for CanoScan LiDE 220 (Canon, Tokyo, Japan)
  ○ Fiji (Schindelin et al., 2012)/ImageJ (Schneider et al., 2012)
  ○ R (R Core Team, 2022)
  ○ RStudio (RStudio Team, 2020) to help to run R

1. **Seed preparation**
  a. Start by separating the seeds from the litter. A sieve with 1‐mm mesh can be helpful here.
  b. If filled and unfilled seeds are to be distinguished, this must be done before the analysis (follow 1bi and 1bii). If not, go to 1c.
    i. The seeds of two of our species (*Thuja plicata* [THPL] and *Callitropsis nootkatensis* [CANO]) cannot be easily tested to determine whether they are filled or unfilled, so they must be manually assessed. They should be separated from the rest of the seeds and labeled with the site name, trap number, date of collection, species name, and status (filled or unfilled) (e.g., AE10, T9, 06.07.2023, THPL, unfilled).
    ii. For the seeds of other species, use a beaker wide enough to fit a tea strainer, label it with the site name, trap number, and date of collection. Soak the seeds in water for at least 72 h, stirring once or twice a day. The seeds that float are considered unfilled and the ones that sink are filled. The floating seeds are collected with a tea strainer, while the remaining seeds are collected from the water using a sieve. Place filled or unfilled seeds in a plastic container, and dry as described in 1c. Label the plastic container with site name, trap number, date of collection, and status (filled or unfilled).
  c. Dry the seeds for 24 h at 40°C.

2. **Scanning the seeds**
  Before scanning, ensure your scanner is set up. Connect the scanner to the computer, open the software, and select the correct scanner.
  a. Distribute the seeds on the scanner surface. They should not touch each other or the edge. When the sample has a large number of seeds, separate them into multiple scans (labeling according to 2c). Seeds from one trap can be distributed over several scans, but each scan must only contain seeds from the same trap and collecting season. Scan each sample twice, mixing up the seeds in between (see 2e).
  b. In the Canon IJ Scan Utility program, go to “Settings” and select to scan as a photo in black‐and‐white mode using the whole scanner surface at 600 dpi. The scan name should follow the naming system detailed under step 2c and be saved as a TIFF to the folder “Scans”, which is a subfolder of “Seed_Analysis” and should not contain images that have already been analyzed, as seen in step 3.
  c. Naming system (to be entered under “Dateiname”):
    Project‐Site‐TrapNumber_Date of collection_Operator_filled/unfilled_PartOfSample_MixNumber
    **Example:** MTR‐TA01‐6_18‐08‐2022_JR_unf_a_m2
    **Project:** MTR for Mt. Rainier and LWF for the Swiss sites
    **Site and trap number:** This should be written on the seed bag. The site code is four characters, and the trap number is a numeric value.
    **Date of collection:** dd‐mm‐yyyy
    **Operator:** Two‐letter code identifying who scanned the seeds: first letter of first name, first letter of last name
    **Filled/unfilled:** Three‐letter code: “unf” for unfilled seeds, “fil” for filled seeds.
    **Part of sample:** A sample with a large number of seeds may have to be split into several scans as per 2a. The first scan is always labeled “a”; if more are needed, proceed with “b”, “c”,…
    **Mix:** Start with “m1” when scanning a batch for the first time. When the seeds are rescanned (see 2e), the scan name will be the same except that the mix will be designated “m2”.
  d. Click OK, then click the icon “Photo”; make sure the lid of the scanner stays closed.
  e. When opening the lid, ensure no seeds are lost. Move them around on the scanner surface, altering their placement, orientation, and which side faces upwards. Scan them again (the name should now end in m2). A minimum of two scans per sample should be performed.
3. **Analyzing the scans**
  Use ImageJ to perform the measurements for the seed species identification. The number of images from the “Scans” folder that can be run simultaneously using the following code depends on the allocated RAM of the computer, although 60–80 is a guideline. Step 3c explains how to check if all scans could be analyzed.
  a. Open the ImageJ/Fiji macro “measuring_bw.ijm”. If asked which program should be used to open the file, choose Fiji.
  b. Click on “Run”, then choose the folder “Scans” containing the scans. If an error occurs when opening the file, save the folder “Scans” to the desktop and try again.
  c. Make sure the program finishes running through all scans and says “command finished” (not “aborted” or “out of memory”). An “out of memory” error would result in a log report as shown in Figure A1. You can also check the filename of the last scan that was analyzed and the last scan in the folder. If the program did not run through all scans, repeat the process on the leftover scans by running the code on a folder containing only the remaining files.
  d. The CSV file with the measurements will be in the same folder as the scans.
  e. To close the open scans, quit ImageJ entirely and **do not save the changes**!
  f. Drag the “Measurements.csv” file from the subfolder “Scans” to the general folder “Seed_Analysis”.
  g. Open the R file “Seed_Classification.R” in RStudio.
  h. Set your directory to the correct folder containing the “Measurements.csv” file (“Seed_Analysis”). You can either type it out in the first row or go via “Session” > “Set working directory” > “Choose directory”, as shown in Figure A2.
  i. Run the entire code (press control + shift + enter or select all of it and click “run”).
  j. Retrieve the file “summary.csv” from the folder “Seed_Analysis” and save it.
  k. Retrieve the file “Measurements.csv” from the folder “Seed_Analysis” and save it.
  l. Remove the scans from the folder “Scans” and save them.

**Notes on running the color version:**

For the colored analysis, “Macro_color_padded_com.ijm” has to be run first in order to cut the scan into images with one seed on each. The measurements can then be obtained with “Measurements_from_padded_color_com.ijm” and the color statistics with “Whole_Color_Macro_com.ijm”. The analysis can be performed using the R file “Seed_Classification_Color_com.R”.

**Notes on applying this to a different data set:**

Ensure that enough samples of every species as well as all locations are present in the training data set. Location names need to be written in exactly the same way (letters and numbers) in the training data set as in the scan filenames.

**Notes on adding other predictor variables:**

If additional predictor variables are identified from the Fiji ImageJ measurements, proceed as in the example using color measurements. If the variables are known from the collection (e.g., season), they should be included in the scan name and processed according to the same procedure we use on site. If the variables are measurements from a different program, it is likely easiest to process them separately until they are imported into R. Measurements could then be matched by scan names. For this, it might be helpful to run the analysis on cropped scans with individual seeds, as we do in the color version, so the measurements of the individual seeds in one scan are matched correctly.
