# Supplementary material for: Seed classification with random forest models
Source: Appl Plant Sci. 2024 Jun 5;12(3):e11596. doi: 10.1002/aps3.11596 (PMC11192156; doi:10.1002/aps3.11596)
Supplement: Supplementary file 1 — Appendix S1. Species names and abbreviations. Appendix S2. Sample size. Appendix S3. Additional classification approaches explored. Appendix S4. Random forest model parameter tuning. Appendix S5. Confusion matrices and node importance of the black‐and‐white model. [file APS3-12-e11596-s001.docx]

**Supporting information for “Seed classification with random forest models”**

**Appendix S1.** Species names and abbreviations.

| **Four-letter abbreviation** | **Full species name** |
| --- | --- |
| ABAM | *Abies amabilis* Douglas ex J. Forbes (Pinaceae) |
| CANO | *Callitropsis nootkatensis* (D. Don) Oerst. (Cupressaceae) |
| PSME | *Pseudotsuga menziesii* (Mirb.) Franco (Pinaceae) |
| THPL | *Thuja plicata* Donn ex D. Don (Cupressaceae) |
| TSHE | *Tsuga heterophylla* Sarg. (Pinaceae) |
| TSME | *Tsuga mertensiana* (Bong.) Carrière (Pinaceae) |

**Appendix S2.** Sample size.

Sample size determined as: number of individual seed scans = number of seeds × 2 scans = number of observations for the models.

|  | ABAM | CANO | PSME | THPL | TSHE | TSME |
| --- | --- | --- | --- | --- | --- | --- |
| Black-and-white model sample size | 75 | 426 | 197 | 2012 | 2860 | 178 |

**Appendix S3.** Additional classification approaches explored.

**Including color as a predictor**

As an alternative to the main black-and-white model, we also ran the random forest (RF) model including color measurements as additional predictors. This takes more time (color scanning and extracting the additional measurements) but may improve the classification results. We provide all the code (Fiji ImageJ macro [based on Strock, 2021], as well as R code) needed to implement this method. In our case, the test accuracy could be slightly improved to 94.87% (95% confidence interval [CI] = 0.931, 0.9629; kappa = 0.9169); however, it is important to note that, especially if seeds were subject to weathering, including color measurements may introduce bias through discoloration (e.g., from lichen or fungal colonization). The training data set should be carefully adapted, and the applicability must be checked.

**Quadratic discriminant analysis**

The quadratic discriminant analysis (QDA) is a supervised machine learning approach in which the model looks for the division (can be a quadratic line) that best divides the different groups onto different ends of the canonical axes (one per *n* – 1 classes) (Hastie et al., 2009). We implemented QDA using the R package MASS (Venables and Ripley, 2002), running it on the measurements presented in Table 1 (like the chosen RF model); however, initial classification results were not promising, and we abandoned this approach in favor of the RF method.

**Deep learning models**

We built TensorFlow (Abadi et al., 2015) deep learning models based on dense layers and convolutional layers for an attempt at image-based classification. Instead of feeding the model measurements per seed as in the RF and QDA approach, we instead cropped individual images from each seed in the scan (the Fiji ImageJ macro for this step is provided) and ran the classification on those images directly. TensorFlow is a so-called “blackbox model” because we do not know what happens during the classification. The user can specify input (the images) and output (six classes) and the number and size of layers in between. On these layers, the model will perform computations in an attempt to best replicate the classification of the training data received. The models were implemented through Google Collaboratory (https://colab.research.google.com/). Attempts to develop a reliable deep learning classifier failed, resulting in the exclusive prediction of the most abundant class or a subset of the six classes.

**Appendix S4.** Random forest model parameter tuning.

**Models to tune**

Two parameters of the random forest (RF) model were tuned: “ntree” (number of trees in the RF) and “mtry” (number of variables randomly sampled as candidates at each split). The full RF models were tuned using all predictors according to Table 1, as was a version excluding all *xy*-type predictors (model containing the predictors in Table 1 excluding X, Y, XM, YM, BX, BY, FeretX, and FeretY), as these have a low node importance in the full model and are less directly meaningful in referring to scan coordinates. The RF models were performed with the function “randomForest” from the R package randomForest (Liaw and Wiener, 2002).

**Tuning for “ntree”**

We started with the default for “ntree” that the randomForest function provides (in this case, 500 for both models) and varied it in both directions. While a higher number of trees generally improved accuracy, the difference was negligible (less than 1% accuracy when varying “ntree” stepwise between 100 and 2500, as detailed in the tables below). We thus settled on a value of “ntree” = 500 as an economic choice, balancing the calculation speed and little accuracy gain from elevating it. For the calculation, we used the R package caret (Kuhn, 2008), specifically the function “train”. Three repetitions and 10-fold cross-validation were applied to the full data set to increase the robustness of the estimates. “mtry” was held constant at the default value that the randomForest function had employed.

After tuning “mtry” as detailed below, we found that, for the model excluding the *xy*-type nodes, an “mtry” value of 6 performed better than the default 4. We repeated the “ntree” tuning for that model again, holding “mtry” constant at 6, and observed no difference in the result.

Full model (mtry = 5 [default and best]):

| **Ntree** | **Accuracy (mean)** | **Kappa (mean)** |
| --- | --- | --- |
| 100 | 0.9222904 | 0.8734524 |
| 200 | 0.9212679 | 0.8726923 |
| 500 | 0.9212679 | 0.8717148 |
| 1000 | 0.9212679 | 0.8721796 |
| 1500 | 0.9222904 | 0.8731795 |
| 2000 | 0.9222904 | 0.8734058 |
| 2500 | 0.9222904 | 0.8735702 |

Excluding *xy*-type nodes (mtry = 4 [default]):

| **Ntree** | **Accuracy (mean)** | **Kappa (mean)** |
| --- | --- | --- |
| 100 | 0.9232354 | 0.8755650 |
| 200 | 0.9232354 | 0.8747856 |
| 500 | 0.9202454 | 0.8703380 |
| 1000 | 0.9212697 | 0.8721703 |
| 1500 | 0.9221311 | 0.8728670 |
| 2000 | 0.9222108 | 0.8731237 |
| 2500 | 0.9222108 | 0.8732952 |

Excluding *xy*-type nodes (mtry = 6 [best]):

| **Ntree** | **Accuracy (mean)** | **Kappa (mean)** |
| --- | --- | --- |
| 100 | 0.9263804 | 0.8800333 |
| 200 | 0.9252824 | 0.8788055 |
| 500 | 0.9232354 | 0.8752217 |
| 1000 | 0.9232354 | 0.8755633 |
| 1500 | 0.9232354 | 0.8752473 |
| 2000 | 0.9223697 | 0.8745446 |
| 2500 | 0.9233147 | 0.8757847 |

**Tuning for “mtry”**

We started with the default for “mtry” that the randomForest function provides (in this case 5 for the full model and 4 for the model excluding *xy*-type nodes) and varied it in both directions until we observed a continuous drop in accuracy. For this, we used the normal randomForest function, keeping “ntree” at 500 and varying “mtry”. The model was trained and tested according to our methods for different values of “mrty”, and accuracy and kappa are reported below. This indicated an optimal value for “mtry” of 5 in the full model and 6 in the model excluding *xy*-type nodes.

Full model (ntree = 500):

| **mtry** | **Accuracy** | **95% CI of accuracy** | **Kappa** |
| --- | --- | --- | --- |
| 3 | 0.9208 | 0.9007, 0.938 | 0.8704 |
| 4 | 0.9267 | 0.9071, 0.9432 | 0.8808 |
| 5 | 0.9313 | 0.9123, 0.9473 | 0.8887 |
| 6 | 0.9267 | 0.9071, 0.9432 | 0.8812 |
| 7 | 0.9255 | 0.9059, 0.9422 | 0.8792 |

*Note:* CI = confidence interval.

Excluding *xy*-type nodes (ntree = 500):

| **mtry** | **Accuracy** | **95% CI of accuracy** | **Kappa** |
| --- | --- | --- | --- |
| 3 | 0.922 | 0.902, 0.939 | 0.8731 |
| 4 | 0.9255 | 0.9059, 0.9422 | 0.879 |
| 5 | 0.9267 | 0.9071, 0.9432 | 0.881 |
| 6 | 0.9302 | 0.911, 0.9463 | 0.8868 |
| 7 | 0.9267 | 0.9071, 0.9432 | 0.8816 |
| 8 | 0.929 | 0.9097, 0.9452 | 0.8852 |
| 9 | 0.929 | 0.9097, 0.9452 | 0.8852 |

**Appendix S5.** Confusion matrices and node importance of the black-and-white model.

Confusion matrix of the prediction of the training data set (prediction in column, reference in row).

|  | ABAM | CANO | PSME | THPL | TSHE | TSME |
| --- | --- | --- | --- | --- | --- | --- |
| ABAM | 64 | 0 | 0 | 0 | 0 | 0 |
| CANO | 0 | 363 | 0 | 0 | 0 | 0 |
| PSME | 0 | 0 | 168 | 0 | 0 | 0 |
| THPL | 0 | 0 | 0 | 1711 | 0 | 0 |
| TSHE | 0 | 0 | 0 | 0 | 2431 | 0 |
| TSME | 0 | 0 | 0 | 0 | 0 | 152 |

Confusion matrix of the prediction of the validation data set (prediction in column, reference in row).

|  | ABAM | CANO | PSME | THPL | TSHE | TSME |
| --- | --- | --- | --- | --- | --- | --- |
| ABAM | 9 | 0 | 0 | 0 | 0 | 0 |
| CANO | 0 | 61 | 0 | 0 | 0 | 0 |
| PSME | 1 | 0 | 20 | 3 | 0 | 0 |
| THPL | 0 | 0 | 9 | 277 | 13 | 0 |
| TSHE | 1 | 0 | 0 | 20 | 411 | 4 |
| TSME | 0 | 2 | 0 | 1 | 5 | 22 |

Node importance (Gini coefficient). Measurements defined in Table 1.

|  | MeanDecreaseGini |
| --- | --- |
| Area | 297.36173 |
| X | 27.69933 |
| Y | 29.42249 |
| XM | 27.24974 |
| YM | 29.88533 |
| Perim. | 194.36458 |
| BX | 27.20298 |
| BY | 30.20232 |
| Width | 51.63959 |
| Height | 58.28998 |
| Major | 124.14711 |
| Minor | 245.09994 |
| Angle | 31.22604 |
| Circ. | 140.48049 |
| Feret | 132.21850 |
| IntDen | 228.13547 |
| Median | 0.00000 |
| RawIntDen | 237.05984 |
| FeretX | 27.72042 |
| FeretY | 29.45838 |
| FeretAngle | 30.26993 |
| MinFeret | 220.29424 |
| AR | 81.66539 |
| Round | 83.45377 |
| Solidity | 93.66374 |
| Site | 562.52468 |

**REFERENCES**

Abadi, M., A. Agarwal, P. Barham, E. Brevdo, Z. Chen, C. Citro, G. S. Corrado, et al. 2015. TensorFlow: Large-scale machine learning on heterogeneous systems. Website <http://tensorflow.org> [accessed 26 April 2024].

Hastie, T., R. Tibshirani, and J. H. Friedman. 2009. The elements of statistical learning: Data mining, inference, and prediction. Springer, New York, New York, USA.

Kuhn, M. 2008. Building predictive models in R using the caret package. *Journal of Statistical Software* 28(5): 1–26.

Liaw, A., and M. Wiener. 2002. Classification and Regression by randomForest. *R News* 2: 18–22. Available at website [https://CRAN.R-project.org/doc/Rnews/](https://cran.r-project.org/doc/Rnews/) [accessed 26 April 2024].

Strock, C. 2021. Protocol for extracting basic color metrics from Images in ImageJ/Fiji. Zenodo. Website <https://doi.org/10.5281/zenodo.5595203> [accessed 26 April 2024].

Venables, W. N., and B. D. Ripley. 2002. Modern Applied Statistics with S, 4th ed. Springer, New York, New York, USA. Website <https://www.stats.ox.ac.uk/pub/MASS4/> [accessed 26 April 2024].
